# Supplementary material for: Impaired prosaposin lysosomal trafficking in frontotemporal lobar degeneration due to progranulin mutations
Source: Nat Commun. 2017 May 25;8:15277. doi: 10.1038/ncomms15277 (PMC5477518; doi:10.1038/ncomms15277)
Supplement: Supplementary Information — Supplementary Figures and Supplementary Table [file ncomms15277-s1.pdf]

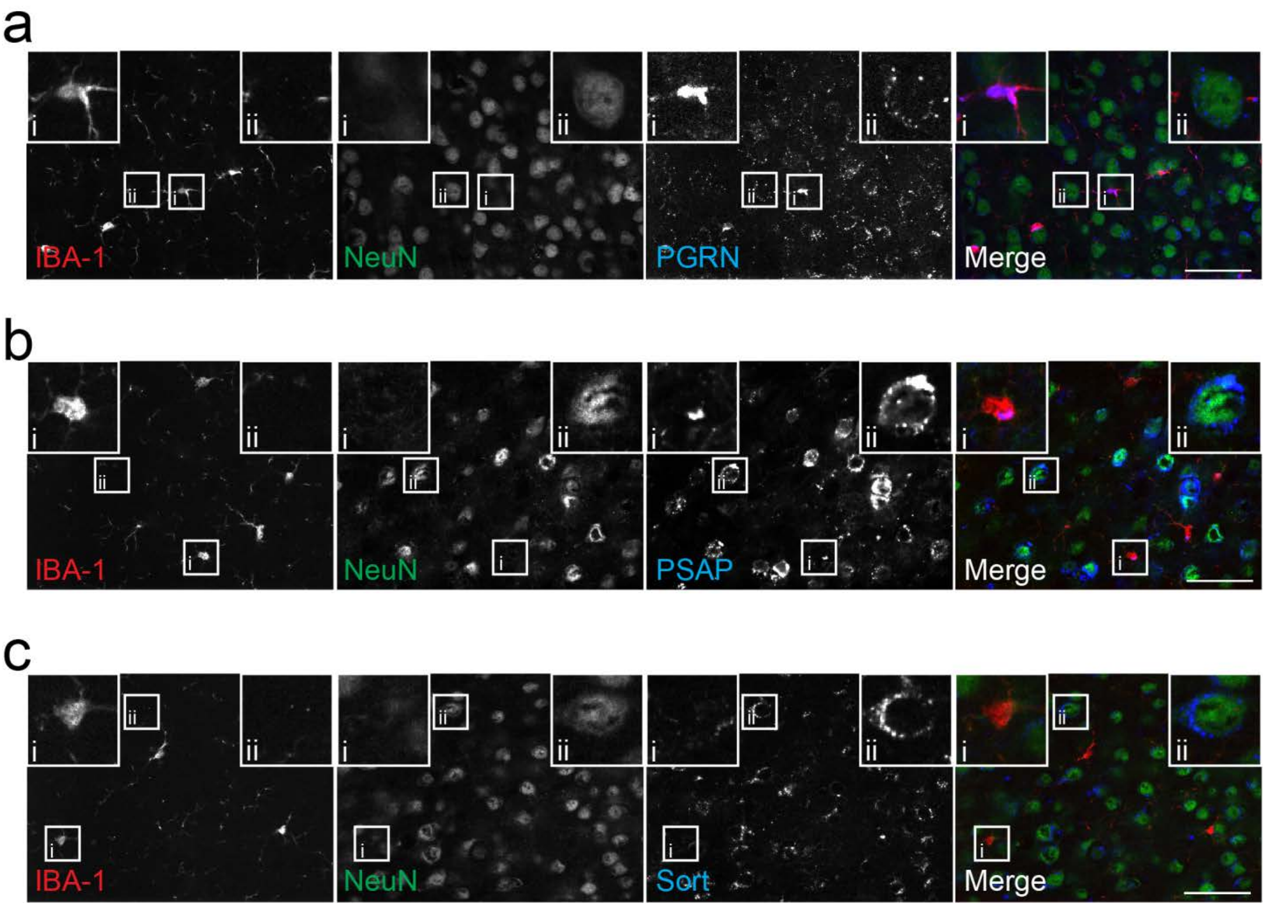


**Supplementary Figure 1. Expression pattern of PGRN, PSAP, and sortilin in normal mouse brain.**

Brain sections from 6 month old WT mouse were stained with anti-IBA1 (marker for microglia), anti-NeuN (marker for neurons) and anti-PGRN (a), PSAP (b) or sortilin (c) antibodies as indicated. Representative image of microglia was shown in inset i and neuron was shown in inset ii. Scale bar=50µm. (a)-(c) are the representative images from two mouse brains.

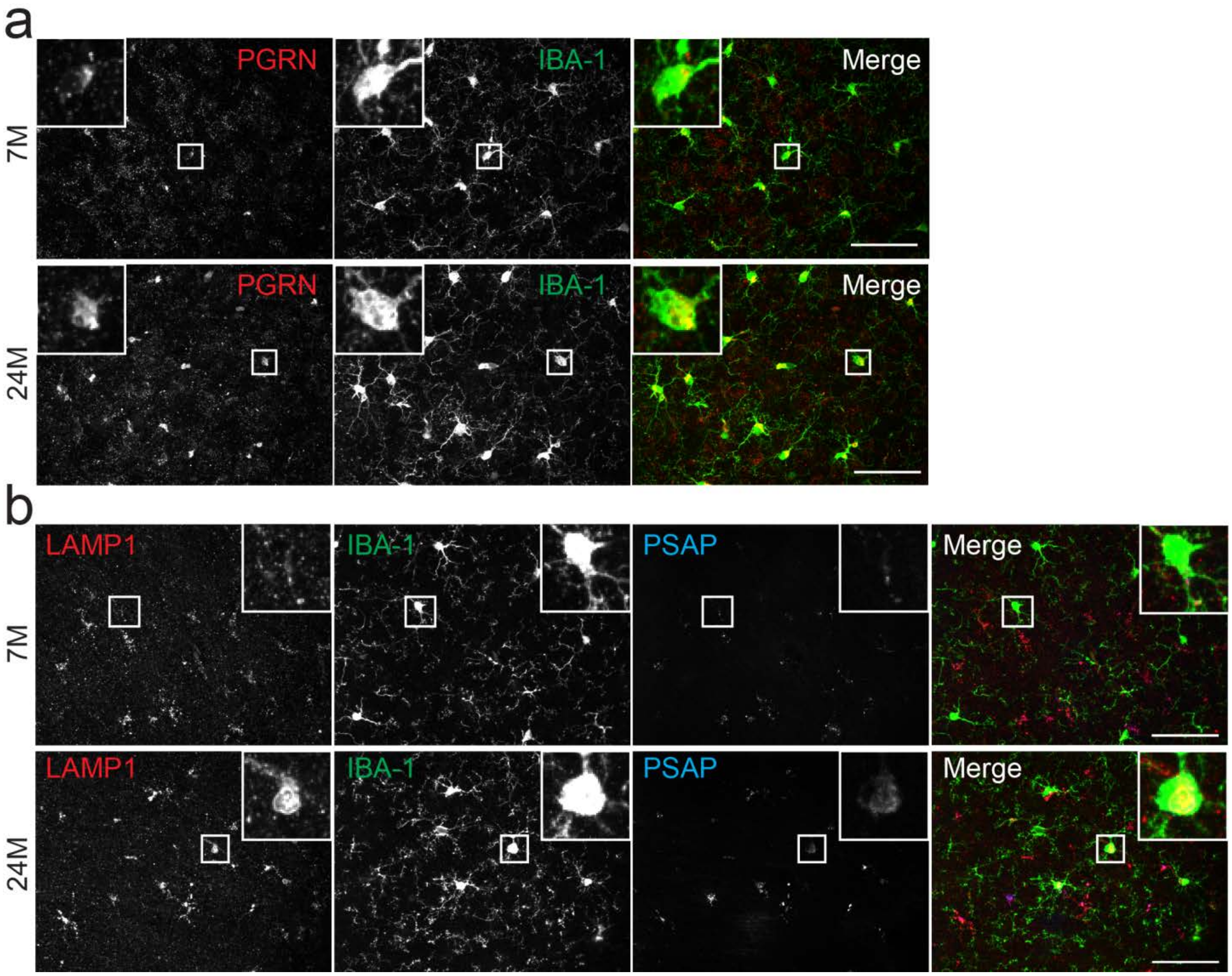


**Supplementary Figure 2. Increased levels of PGRN and PSAP in activated glia during normal aging.**

(a) Brain sections from 7 or 24 month old WT mouse were stained with sheep anti-PGRN and rabbit anti-IBA-1 antibodies. Representative image of microglia was shown in inset. Scale bar=50μm.
(b) Brain sections from 7 or 24 month old WT mouse were stained with rat anti-LAMP1, rabbit anti-IBA-1 and goat anti-PSAP antibodies. Representative image of microglia was shown in inset. Elevated levels of PSAP are also seen in other cell types, likely activated astrocytes during aging. Scale bar=50μm. (a) and (b) are the representative images from three mouse brains.

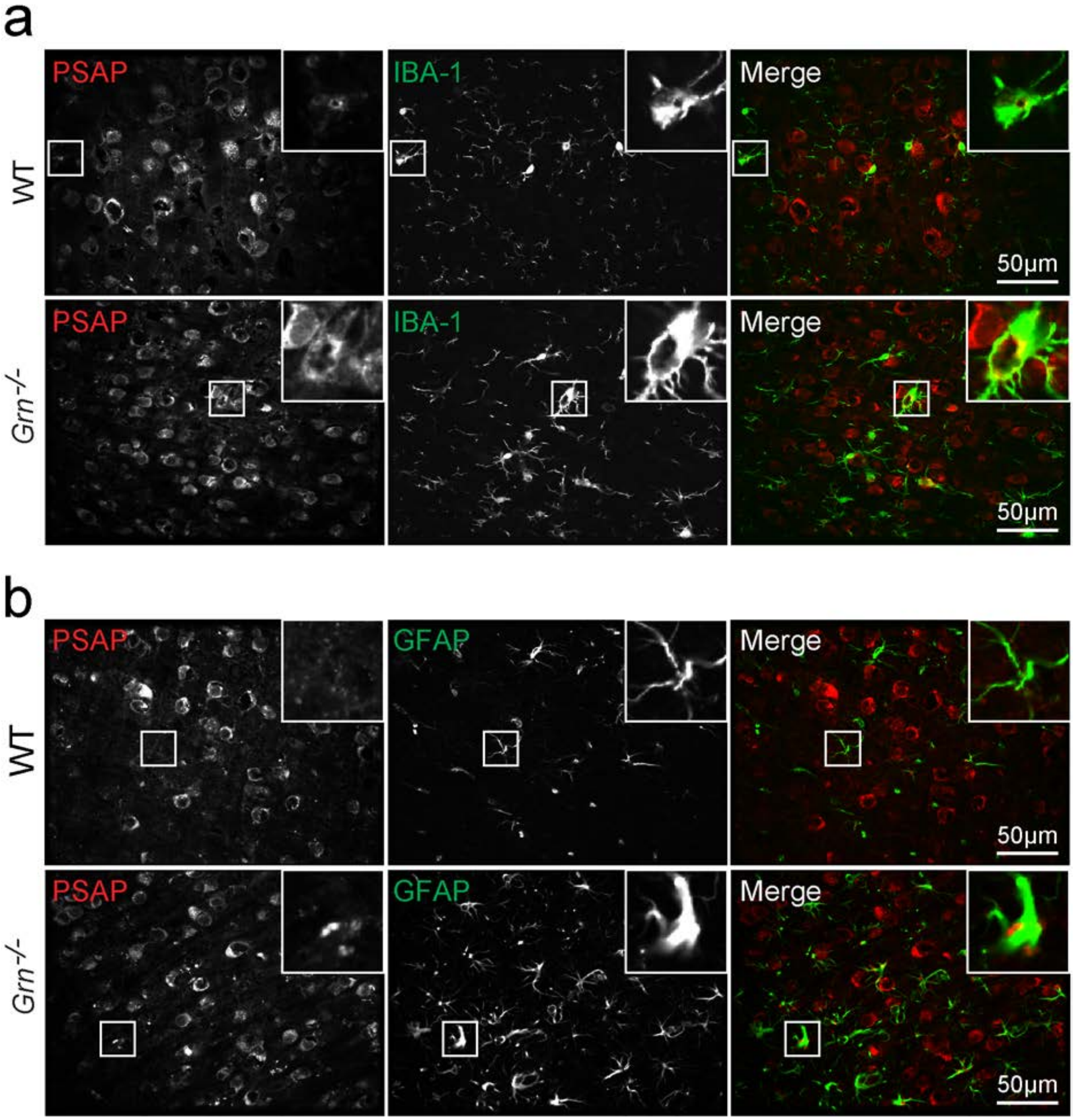


**Supplementary Figure 3. Glial PSAP levels are increased in *Grn*$^{-/-}$ mouse brain.**

(a) Representative immunostaining images of 12-14 month old brain sections of WT and *Grn*$^{-/-}$ mice with goat anti-PSAP, and rabbit anti-IBA-1 antibodies. Representative image of IBA-1 positive microglia was shown in inset. (b) Representative immunostaining images of 12-14 month old brain sections of WT and *Grn*$^{-/-}$ mice with rabbit anti-PSAP, and mouse anti-GFAP antibodies. Representative image of GFAP positive astroglia was shown in inset. (a) and (b) are the representative images from three pairs of mouse brains.

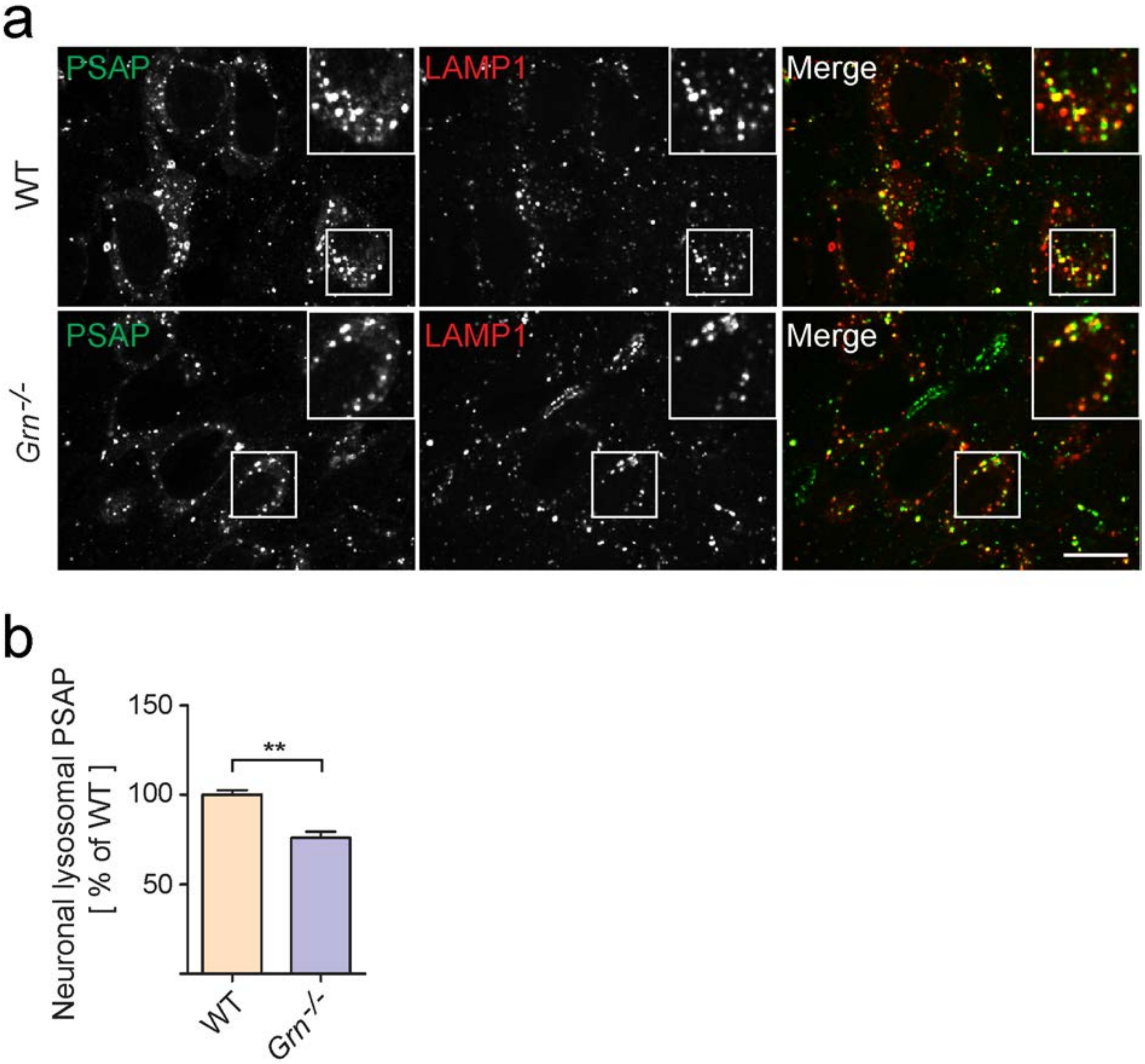


**Supplementary Figure 4. PGRN deficient mice display reduced lysosomal PSAP in neurons.** (a) Representative images from immunostaining of 12-14 month old brain sections of WT and *Grn*$^{-/-}$ mice with anti-mouse PSAP and LAMP1 antibodies. Scale bar=10µm. (b) Quantification of immunofluorescence intensity of lysosomal PSAP in neurons for images in (a), n=4, **, p<0.01, Student's t-test

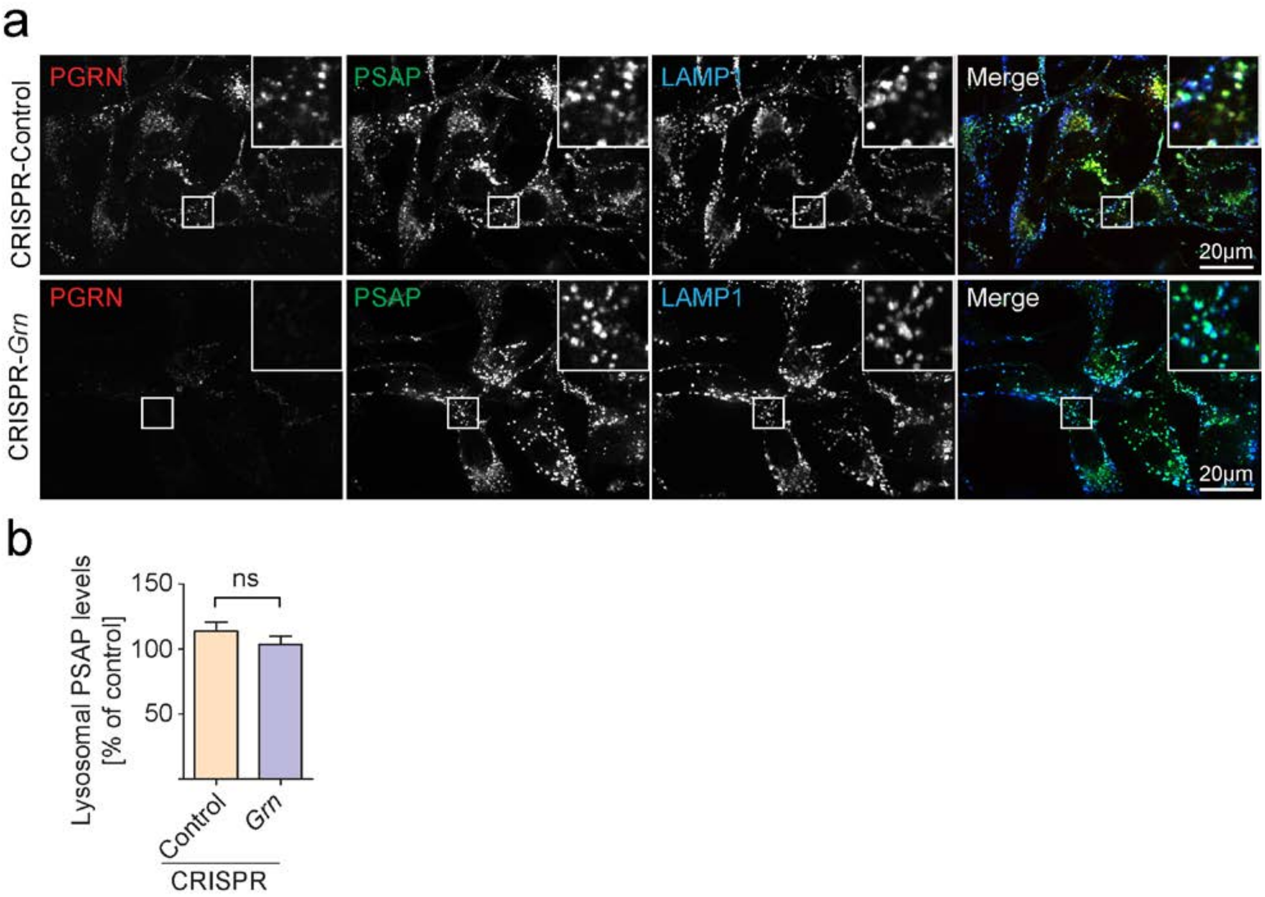


**Supplementary Figure 5. PGRN is not required for lysosomal trafficking of PSAP from biosynthetic pathway in N2a cells.**

(a) *Grn*$^{-/-}$ (CRISPR-*Grn*) or control (CRISPR-Control) N2a cells were stained with sheep anti-PGRN, rabbit anti-PSAP, and rat anti-LAMP1 antibodies. Representative image of PSAP localization with LAMP1 was shown in insets. Scale bar=20μm. (b) Quantification of lysosomal PSAP signals in *Grn*$^{-/-}$ (CRISPR-*Grn*) or WT (CRISPR-Control) cells in (a) using Image J. n=42; ns, not significant; Student's t-test.

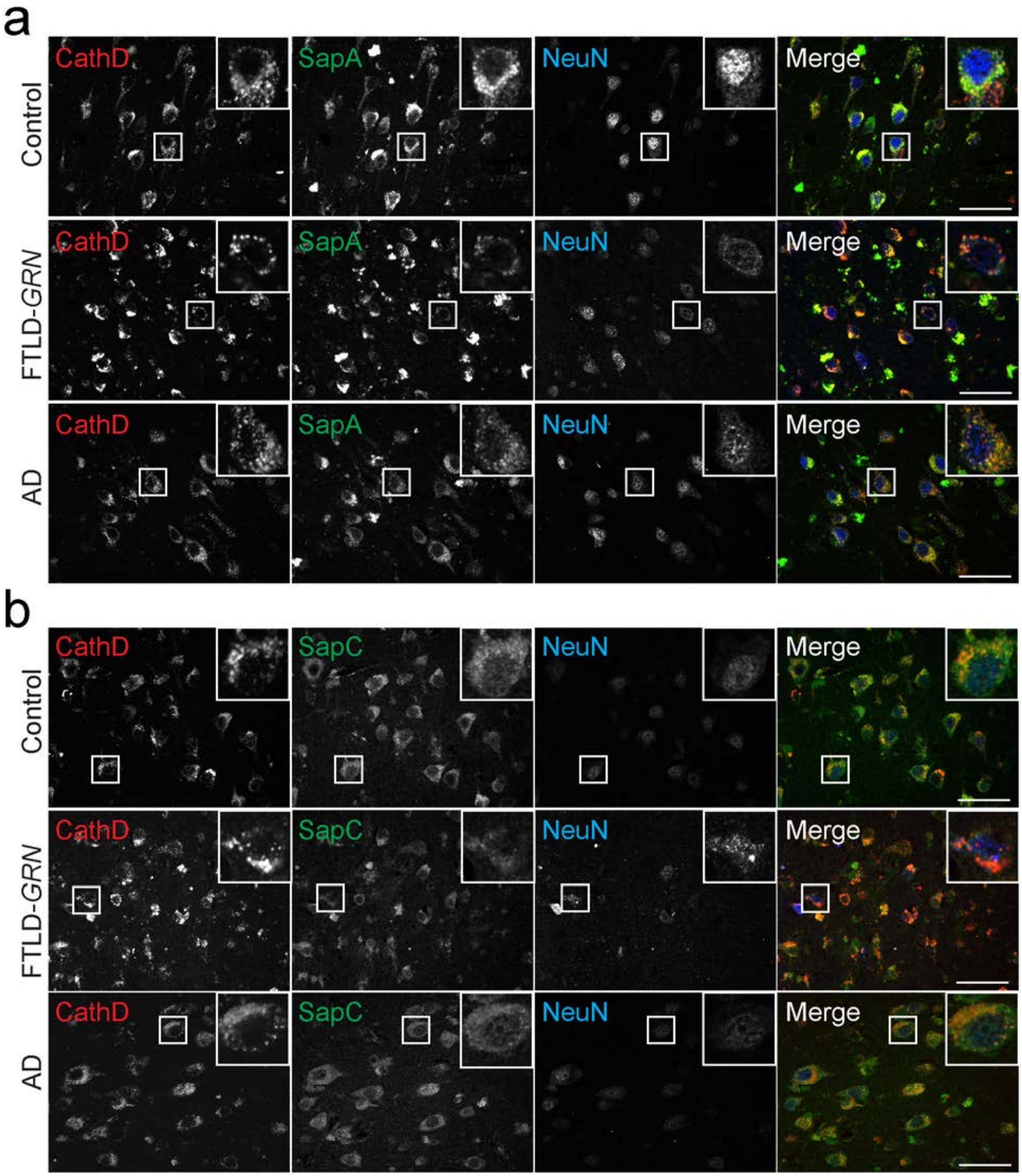


**Supplementary Figure 6. Reduced saposin A and saposin C levels in neurons in patients with FTLD due to *GRN* mutations.**

(a) Brain sections from control, FTLD-*GRN* and AD patients were stained with goat anti-cathepsin D (CathD), rabbit anti-saposin A (SapA) and mouse anti-NeuN (marker for neuron) antibodies. Scale bar=50 μm.(b) Brain sections from control, FTLD-*GRN* and AD patients were stained with goat anti-cathepsin D (CathD), rabbit anti-saposin C (SapC) and mouse anti-NeuN (marker for neuron) antibodies. Scale bar=50 μm. (a) and (b) are the representative images from two brains for each group.

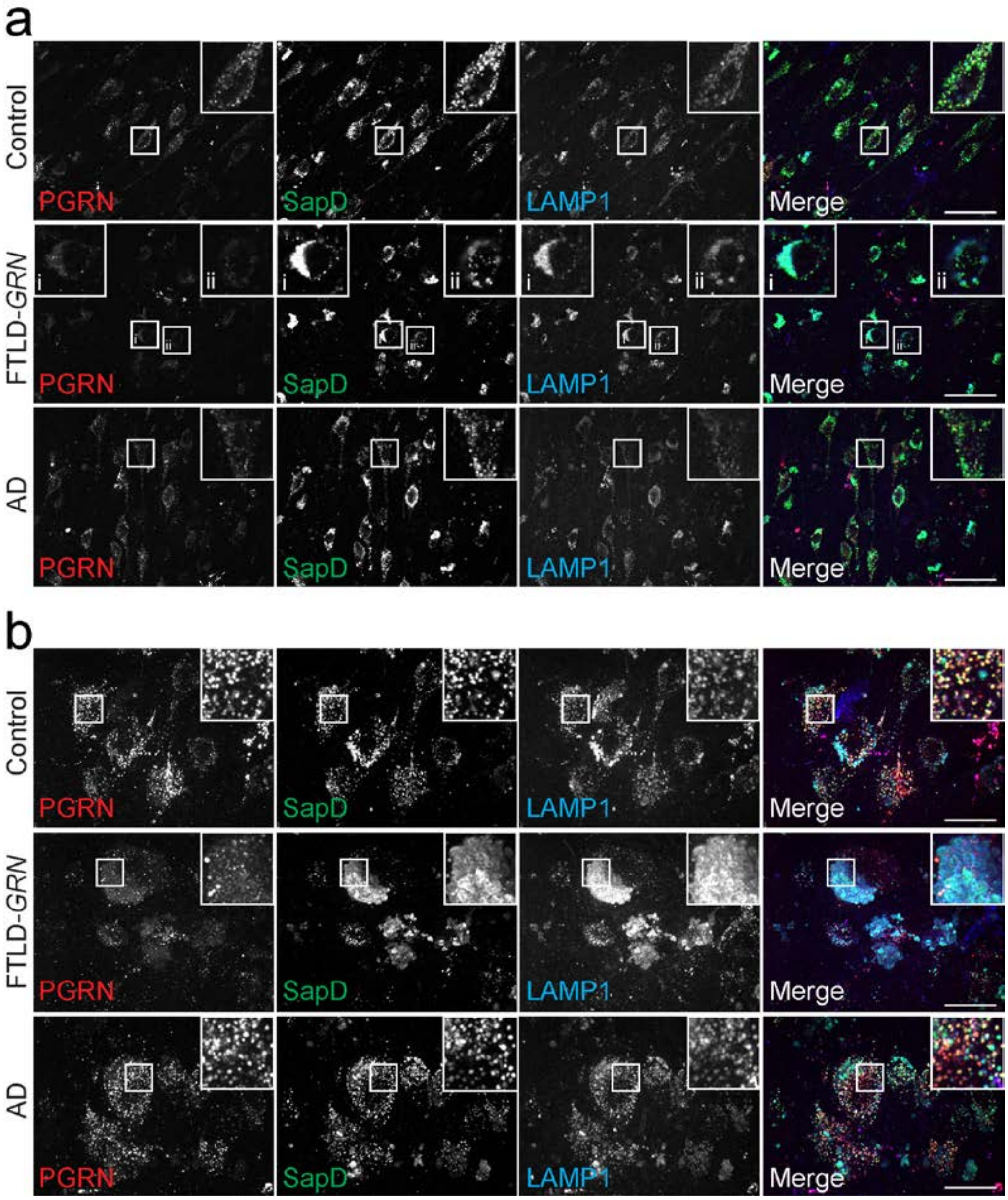


**Supplementary Figure 7. Neuronal saposin D levels in patient brains from control, FTLD due to *GRN* mutations, and AD.**

**(**a**)** Brain sections from control, FTLD-*GRN* and AD patients were stained with sheep anti-PGRN, rabbit anti-saposin D (SapD) and mouse anti-LAMP1 (marker for lysosome) antibodies. Representative images of neurons with enlarged (i) or relative normal (ii) lysosomes were shown in insets. Scale bar=50µm. **(**b**)** Higher magnification of saposin D (Sap D) staining in control, FTLD-*GRN* and AD patients. Representative images of the co-staining of PGRN, SapD, and LAMP1 were shown in inset. Scale bar=20µm. (a) and (b) are the representative images from two brains for each group.

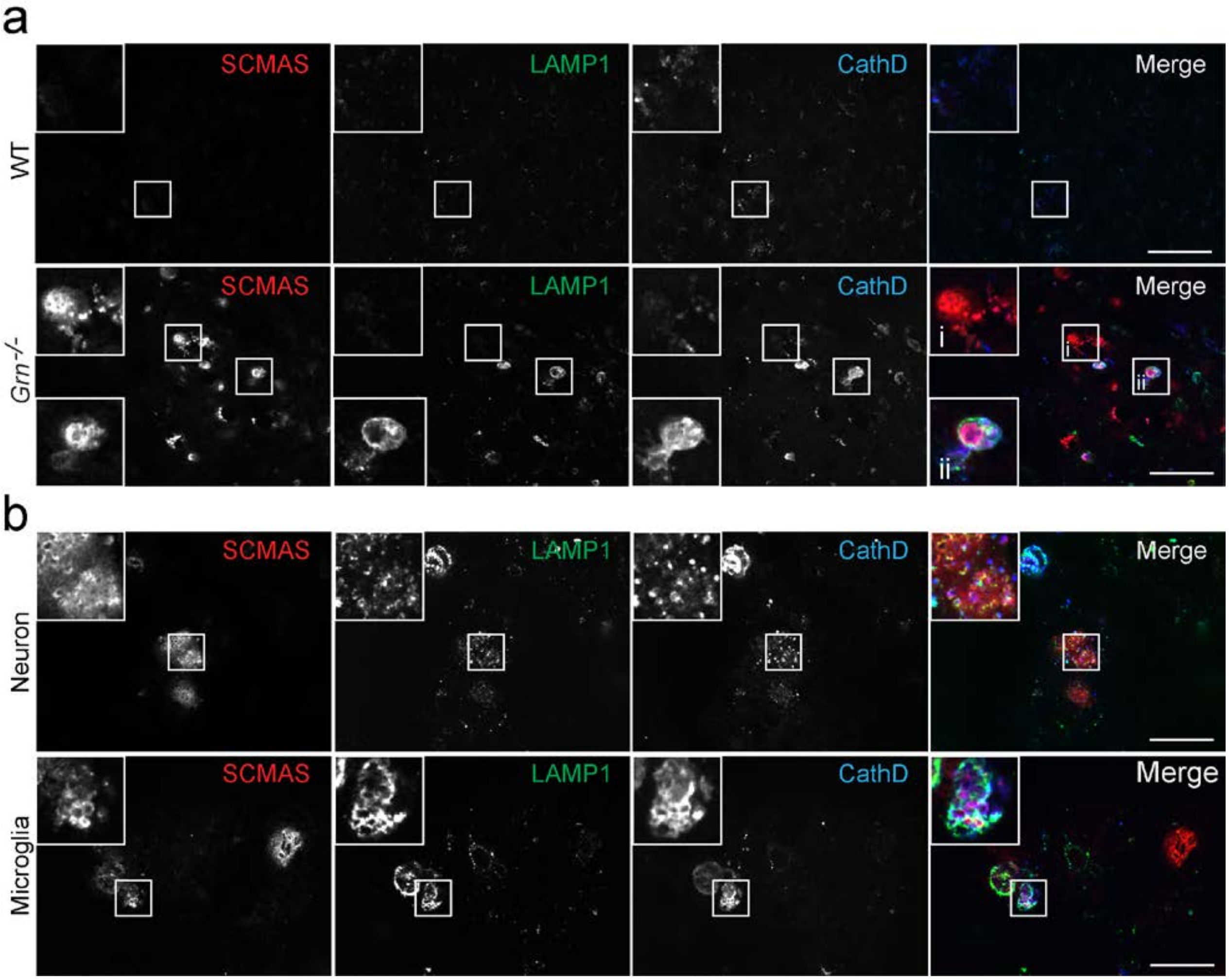


**Supplementary Figure 8. The lipophilic subunit c of the mitochondrial ATP synthase (SCMAS) forms aggregates in both neuron and glia cells in aged *Grn*$^{-/-}$ mouse brain.**

(a) Brain sections from 20 month old WT and *Grn*$^{-/-}$ mice were stained with anti-SCMAS, anti-LAMP1, and anti-cathepsin D (CathD) antibodies. A representative picture of neuron from *Grn*$^{-/-}$ mice was shown in inset i and microglia shown in inset ii. Scale bar=50µm. (b) Higher magnification of mitochondrial ATP synthase accumulated neuron and microglia from 20 month old *Grn*$^{-/-}$ mice brain. Representative images of the co-staining of SCMAS, LAMP1, and cathepsin D were shown in inset. Scale bar=20µm. (a) and (b) are the representative images from two brains for each group.

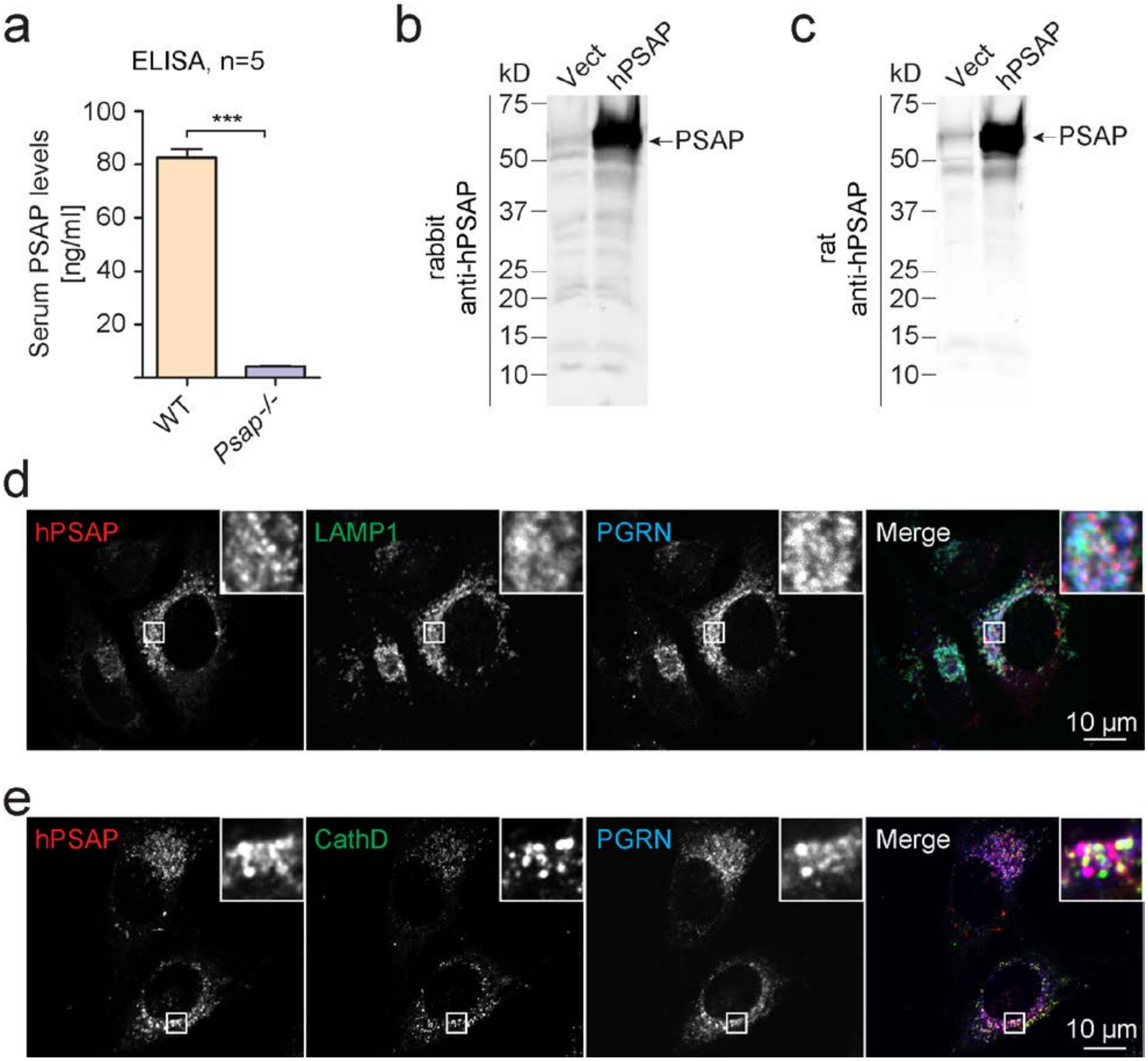


**Supplementary Figure 9. Validation of the specificity of home-made anti-PSAP antibodies.**

(a) Validation of the specificity of rabbit anti-mouse PSAP in ELISA. n=5, ***, p<0.001, Student t-test. (b) and (c) Validation of the specificity of rabbit anti-human PSAP (b) and rat anti-human PSAP (c) antibodies by Western blots. Lysates from HEK293T transfected with vector control or hPSAP expressing constructs were analyzed using anti-human PSAP antibodies as indicated. (d) and (e) Validation of the specificity of rabbit anti-human PSAP (d) and rat anti-human PSAP (e) antibodies in immunofluorescence staining. Hela cells were fixed and immunostained as indicated. Both rabbit and rat anti-human PSAP antibodies show specific staining that overlaps with LAMP1 and PGRN. Scale bar=10 µm.

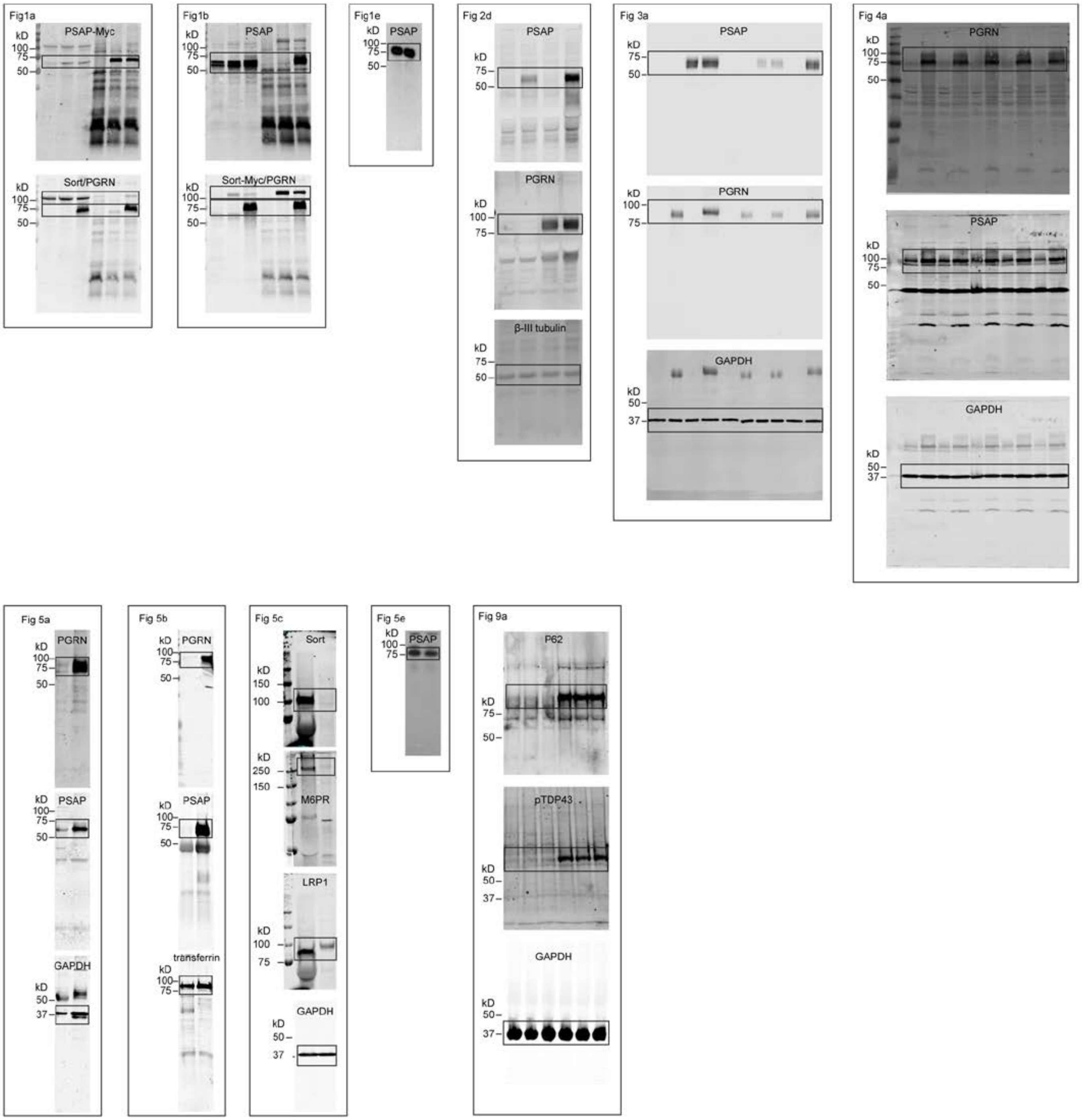


**Supplementary Figure 10. Original full size Western blots**

| Pathology/Brain number | Sex | Age at death (years) | Clinical diagnosis | Primary neuropathological diagnosis | Post-mortem interval (hrs) | Anatomical Region (gyrus) | Regional neuropath score: Vacuolation | Regional neuropath score: Gliosis |
|---|---|---|---|---|---|---|---|---|
| Control 1 | F | 81 | aMCI | Mild neurofibrillary changes (Braak Stage 2) | 30.3 | Anterior orbital gyrus | 0 | 0 |
| Control 2 | F | 86 | Control | Mild cerebrovascular disease | 7.8 | Anterior orbital gyrus | 0 | 0 |
| Control 3 | M | 76 | Control | Low ADNC | 8.2 | Anterior orbital gyrus | 0 | 0 |
| Control 4 | F | 86 | Control | Incidental LBD, brainstem predominant | 6.4 | Anterior orbital gyrus | 0 | 0 |
| FTLD/GRN 1 | M | 74 | PNFA/CBS | FTLD-TDP-A | 30.9 | Anterior orbital gyrus | 1 | 2 |
| FTLD/GRN 2 | F | 66 | bvFTD | FTLD-TDP-A | 7.4 | Anterior orbital gyrus | 2 | 3 |
| FTLD/GRN 3 | M | 68 | bvFTD | FTLD-TDP-A | 13.5 | Anterior orbital gyrus | 3 | 3 |
| FTLD/GRN 4 | M | 64 | bvFTD | FTLD-TDP-A | 7.2 | Anterior orbital gyrus | 2 | 2 |
| AD 1 | M | 84 | AD | AD | 8.2 | Anterior orbital gyrus | 0 | 0 |
| AD 2 | F | 69 | AD | AD | 12.9 | Anterior orbital gyrus | 0 | 0 |
| AD 3 | F | 54 | AD | AD | 16.0 | Anterior orbital gyrus | 0 | 0 |
| AD 4 | M | 70 | AD | AD | 9.0 | Anterior orbital gyrus | 0 | 0 |
| FTLD/Tau 1 | M | 61 | bvFTD | CBD | 5.0 | Anterior orbital gyrus | 0 | 1 |
| FTLD/Tau 2 | M | 63 | bvFTD | CBD | 6.2 | Anterior orbital gyrus | 0 | 0 |
| FTLD/Tau 3 | F | 73 | bvFTD | CBD | 6.6 | Anterior orbital gyrus | 2 | 2 |

**Supplementary Table 1. Summary of pathological, clinical, and genetic information of human brain tissues of FTLD-TDP (*GRN*), FTLD-Tau, AD, and controls.**
